# The impact of preoperative oral nutrition supplementation on outcomes in patients undergoing gastrointestinal surgery for cancer in low- and middle-income countries: a systematic review and meta-analysis

**DOI:** 10.1038/s41598-022-16460-4

**Published:** 2022-07-21

**Authors:** Stephen R. Knight, Ahmad U. Qureshi, Thomas M. Drake, Marie Carmela M. Lapitan, Mayaba Maimbo, Edwin Yenli, Stephen Tabiri, Dhruva Ghosh, Pamela A. Kingsley, Sudha Sundar, Catherine Shaw, Apple P. Valparaiso, Aneel Bhangu, Peter Brocklehurst, Laura Magill, Dion G. Morton, John Norrie, Tracey E. Roberts, Evropi Theodoratou, Thomas G. Weiser, Sorrel Burden, Ewen M. Harrison

**Affiliations:** 1 Centre for Medical Informatics, Usher Institute, Nine Bioquarter, University of Edinburgh, Edinburgh, EH16 4UX UK; 2 Department of Surgery, Services Institute of Medical Sciences, Lahore, Pakistan; 3 Department of Surgery, National Institutes of Health, University of the Philippines, Manila, Philippines; 4 Department of General Surgery, Kitwe Teaching Hospital, Kitwe, Zambia; 5 Department of Surgery, School of Medicine, University for Development Studies, Tamale, Ghana; 6 Dean of School of Medicine, University for Development Studies, Tamale, Ghana; 7 Department of Paediatric Surgery, Christian Medical College, Ludhiana, India; 8 Department of Radiation Oncology Department, Christian Medical College, Ludhiana, India; 9 Institute of Cancer and Genomic Sciences, University of Birmingham, Birmingham, UK; 10 Clinical and Experimental Medicine, University of Birmingham, Birmingham, UK; 11 Institute of Applied Health Research, University of Birmingham, Birmingham, UK; 12 Centre for Global Health, Usher Institute, University of Edinburgh, Edinburgh, UK; 13 Cancer Research UK Edinburgh Centre, Institute of Genetics and Cancer, University of Edinburgh, Edinburgh, UK; 14 Department of Surgery, Stanford University, Stanford, USA; 15 Department of Clinical Surgery, University of Edinburgh, Edinburgh, UK; 16 School of Health Sciences, University of Manchester, Manchester, UK

**Keywords:** Gastrointestinal cancer, Outcomes research

## Abstract

Malnutrition is an independent predictor for postoperative complications in low- and middle-income countries (LMICs). We systematically reviewed evidence on the impact of preoperative oral nutrition supplementation (ONS) on patients undergoing gastrointestinal cancer surgery in LMICs. We searched EMBASE, Cochrane Library, Web of Science, Scopus, WHO Global Index Medicus, SciELO, Latin American and Caribbean Health Sciences Literature (LILACS) databases from inception to March 21, 2022 for randomised controlled trials evaluating preoperative ONS in gastrointestinal cancer within LMICs. We evaluated the impact of ONS on all postoperative outcomes using random-effects meta-analysis. Seven studies reported on 891 patients (446 ONS group, 445 control group) undergoing surgery for gastrointestinal cancer. Preoperative ONS reduced all cause postoperative surgical complications (risk ratio (RR) 0.53, 95% CI 0.46–0.60, P < 0.001, *I*^2^ = 0%, n = 891), infection (0.52, 0.40–0.67, P = 0.008, *I*^2^ = 0%, n = 570) and all-cause mortality (0.35, 0.26–0.47, P = 0.014, *I*^2^ = 0%, n = 588). Despite heterogeneous populations and baseline rates, absolute risk ratio (ARR) was reduced for all cause (pooled effect −0.14, −0.22 to −0.06, P = 0.006; number needed to treat (NNT) 7) and infectious complications (−0.13, −0.22 to −0.06, P < 0.001; NNT 8). Preoperative nutrition in patients undergoing gastrointestinal cancer surgery in LMICs demonstrated consistently strong and robust treatment effects across measured outcomes. However additional higher quality research, with particular focus within African populations, are urgently required.

## Introduction

Malnutrition is a major public health issue in low- and middle-income countries (LMICs) and forms part of the United Nations 2030 Agenda for Sustainable Development^1^. The predominant focus in LMICs has been child nutrition, yet as many as two-thirds of hospitalised adult patients are malnourished in this setting^2^. Malnutrition is associated with higher postoperative mortality and morbidity, including longer length of in-patient stay and increased healthcare-associated costs^2–4^. Furthermore, preoperative nutrition has been identified as an area of high research priority in LMICs^5^.

Provision of safe and equitable surgical care is becoming increasingly recognised as an essential part of cancer care and population health^6^. In the majority of solid tumours, surgery provides the best chance of cure, particularly where chemotherapy and radiotherapy are unavailable^7^. Oral nutritional supplementation (ONS) provided at the time of surgery in LMICs could provide a low-cost and sustainable intervention, requiring minimal specialist training and equipment to administer. Several reviews and meta-analyses have demonstrated the beneficial effects of preoperative nutrition on surgical site infection, peri-operative complication rate and length of stay^8–10^. However, data is lacking from a systematic review of the evidence exploring the impact of standard oral nutritional supplementation an LMIC setting.

This systematic review and meta-analysis investigated the effect of preoperative oral nutrition on postoperative outcomes for patients undergoing gastrointestinal surgery for cancer in LMICs.

## Materials and methods

### Search strategy and selection criteria

The systematic review protocol was registered prospectively with the PROSPERO database (CRD42019125161)^11^. A systematic search of the EMBASE, Cochrane Library, Web of Science, Scopus, WHO Global Index Medicus, SciELO, Latin American and Caribbean Health Sciences Literature (LILACS) databases, together with a grey literature search using Google Scholar, was performed in accordance with the PRISMA guidelines^12^.

Search terms relating to preoperative oral nutritional intervention in patients undergoing surgery for solid tumours were combined with LMIC filters as specified by the Cochrane Collaboration^13^. The following exploded Medical Subject Headings (MeSH) were used: “surgery”, “cancer”, “malignancy”, “nutrition”, “diet” combined with “postoperative outcomes” (Supplementary Material). Databases were searched from inception, with no limits on publication year or language placed. The reference list of all studies that met the inclusion criteria and review articles were searched manually for additional studies. The trial registry clinicaltrials.gov was searched to identify any unpublished studies. The final literature search was performed on 21st March 2022. Non-randomised, retrospective, review articles, letters to the editor, case reports and conference abstracts with no access to the entire study were excluded.

All studies identified were screened independently by two reviewers from a pool of seven (SB, UQ, TMD, CML, MM, EY, SS) using the online systematic review tool Covidence^14^. All disagreements were adjudicated by a third reviewer (SK).

Randomised controlled trials reporting at least one clinical outcome in an LMIC population based on the World Bank classification at the time of study publication^15^ were included, as described previously^16^. Studies were required to be in patients aged 18 years or above undergoing surgery for gastrointestinal cancer, defined as any procedure requiring a skin incision under regional or general anaesthesia. The intervention required the use of an oral nutritional supplement (ONS) containing macronutrients (fat, carbohydrate and protein) with or without micronutrients (vitamins and minerals). The control arm was patients receiving routine care with no additional dietary supplementation. Therefore, the only difference between the intervention and control groups was the additional preoperative intake of ONS.

Studies with a nutritional intervention using single nutrient substrates, complementary food substances, probiotic formulas or as part of a multimodal preoperative intervention (such as an enhanced recovery programme) and those delivered by enteral tubes or parenteral routes were excluded. Those studies that only reported on postoperative administration of a nutritional intervention were also excluded. Additionally, studies that met the inclusion criteria but did not report data separately for malignant and non-malignant surgery, or between patients within a high-income and LMIC setting were also excluded if attempts to obtain the relevant data failed. A summary of inclusion and exclusion criteria is provided in Supplementary Table S1.

### Data extraction and statistical analysis

Non-English articles were translated by medically qualified individuals where appropriate. Data were retrieved from published articles using a standardised data extraction form for all included studies, including publication details, study design, country, participant number, proportion of malnourished participants, cancer type, surgical procedures performed, participant age, oral nutritional intervention used, follow-up period, 30-day complication rate, all-cause mortality and length of stay. Attempts were made to contact study authors if any data were unclear within the published manuscript or study protocol. Assessment of methodological quality was performed for all included studies using the Cochrane Risk-of-Bias tool^17^. Publication bias was assessed through funnel plot symmetry and statistical analysis using Egger’s test for each outcome.

All binary outcome measures were summarised as risk ratios (RRs) with 95% confidence intervals (CIs), with individual study weights calculated for pooled analysis. Risk ratios (RR) were reported in accordance with the Cochrane Collaboration to avoid overestimation of any potential treatment effect^18^. For individual trials with zero event data in one or more groups, a continuity correction of 0.5 was performed to provide a more conservative estimate of effect size^19^. The presence of statistical heterogeneity was expected, due to the in-between study variability in cancer type, geographical setting, malnutrition rate and oral nutrition provided. Therefore, pooled data analyses were performed using the Mantel–Haenszel random-effects model using the R meta package (v3.6.3).

Absolute Risk Reduction (ARR) was used to estimate population and baseline rate heterogeneity for each outcome, excluding those including zero event data^19^, with pooled estimates calculated as previously stated. The number needed to treat to benefit (NNT), estimating the number of patients that need to be treated in order to have an impact on one person, was defined as the reciprocal of the absolute risk reduction.

Influence analyses, using the leave-one-out method, were performed to determine robustness of pooled effect estimates^19,20^ Heterogeneity was assessed using the *I*^2^ statistic and defined as low, moderate or high with the corresponding upper limits of 25%, 50% and 75%, respectively^17^.

### Role of funding source

The study sponsors had no role in study design, data collection, data analysis, data interpretation, or writing of the report. All authors had full access to all the data in the study and had final responsibility for the decision to submit for publication.

### Patient and public involvement statement

Patient representatives for the NIHR Global Health Research Unit on Global Surgery, from both the UK and Rwanda, guided development of the research question.

## Results

### Literature search

**Figure 1:**
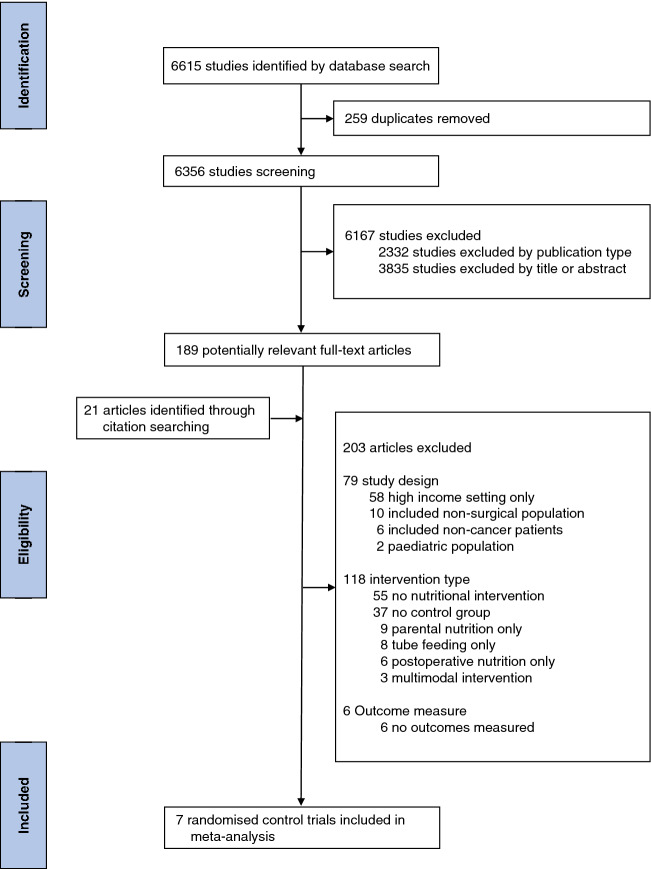
Article selection process.

The systematic search yielded 6615 studies. After the removal of 259 duplicates, 6146 articles were excluded by publication type, or on the basis of title or abstract. Of the remaining 210 articles, 203 did not meet inclusion criteria, with a final seven articles included in the meta-analysis (Fig. 1)^21–27^. The reasons for article exclusion are stated within Fig. 1. A summary of study and patient characteristics are provided in Table 1 and Supplementary Table S2. Six studies originated from China^21–26^ and one from India^27^. All studies included patients undergoing surgery for gastrointestinal malignancy, with four^22–25^ investigating preoperative oral nutrition in gastric cancer surgery only. A total of 891 patients (446 oral nutrition group, 445 control group) were included within the meta-analysis. Six studies reported the recruitment of a full cohort with malnutrition as measured by validated tools (Table 1), with one study not reporting nutritional status^26^.

**Table 1:** Summary of included randomised control trials.

	Year	Country	Cancer type(s)	Patient number Intervention/Control	Patients malnourished (%)	Screening tool used
Wu et al^21^	2006	China	Gastric, colon and rectal cancer	235/233	100	SGA
Ding et al^22^	2009	China	Gastric cancer	21/21	100	NRS-2002
Zheng et al^23^	2010	China	Gastric cancer	18/18	100	NRS-2002
Kharbuja et al^24^	2013	China	Gastric cancer	92/93	100	NRS-2002
Chen et al^26^	2013	China	Rectal cancer	30/30	*ns*	*ns*
Zhou et al^25^	2016	China	Gastric cancer	20/20	100	NRS-2002
Sagar et al^27^	2019	India	Oesophageal and gastric cancer	30/30	100	SGA

*SGA* Subjective Global Assessment, *NRS* Nutritional Risk Screening, *ns* not stated.

### Included study design

A number of oral nutritional formulations were used, each compared to a standard diet control. Nutrison liquid (Nutricia®), commonly given by feeding enteral tube in high-income countries, was used as an oral supplement in three studies^22–24^. Treatment regimen and duration varied between studies, with nutrition commenced for at least five days preoperatively in four studies^21,24,25,27^. Nutritional supplementation characteristics are summarised in Supplementary Table S1.

Patient follow-up to 30 days occurred in all studies, with the majority evaluating postoperative complications as their primary outcome. Some studies additionally reported nutrition status and serum biomarkers as outcomes. Length of stay was not sufficiently reported in three studies for meta-analysis inclusion^21,27^. A summary pooled estimates across all measured outcomes can be found in Supplementary Table S3.

### Outcomes

**Figure 2:**
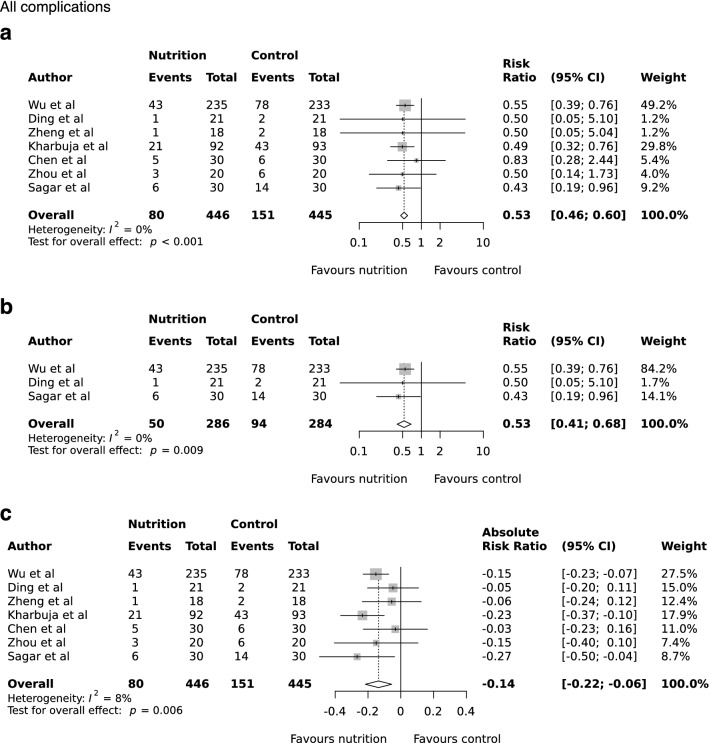
Random-effects meta-analysis of the effects of preoperative oral nutrition on postoperative complications (**a**), when nutritional support was provided for at least 5 days pre-operatively (**b**), and risk difference (**c**) in patients undergoing surgery for gastrointestinal cancer.

All studies provided incident rates for postoperative complications at 30 days. The pooled event rate was 17.9% (80/446) in the oral nutrition group compared with 33.9% (151/445) in the control arm. The pooled RR for complications after preoperative treatment with oral nutrition was 0.53 (95% CI 0.46 to 0.60, P < 0.001, *I*^2^ = 0%; Fig. 2a). This effect persisted when including only studies which recruited malnourished patients (RR 0.53; 95% CI 0.41 to to 0.68, P = 0.009, *I*^2^ = 0%; Fig. 2b). The type of nutrition intervention did not modify the overall effect, with a pooled RR of 0.54 (95% CI 0.40 to 0.73, P = 0.007, *I*^2^ = 0%) following exclusion of studies using Nutrison® liquid (Supplementary Fig. S1). Influence and sensitivity analysis demonstrated the persistence in overall effect for the nutritional supplement group (Supplementary Figs. S2 and S3). The intervention had a consistently strong positive effect despite baseline rate heterogeneity across included studies (ARR range −0.22 to −0.06, pooled effect −0.14, 95% CI −0.22 to −0.06, P = 0.006; Fig. 2c), with a corresponding number needed to treat (NNT) of 7 (95% CI 5 to 17).

**Figure 3:**
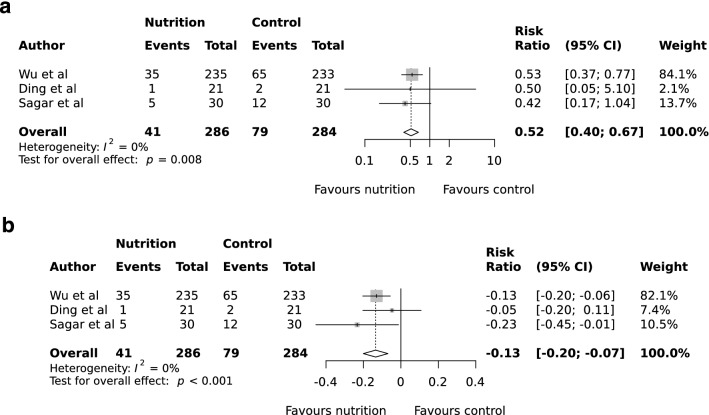
Random-effects meta-analysis of the effects of preoperative oral nutrition on infectious complications (**a**), and risk difference (**b**) in patients undergoing surgery for gastrointestinal cancer.

**Figure 4:**
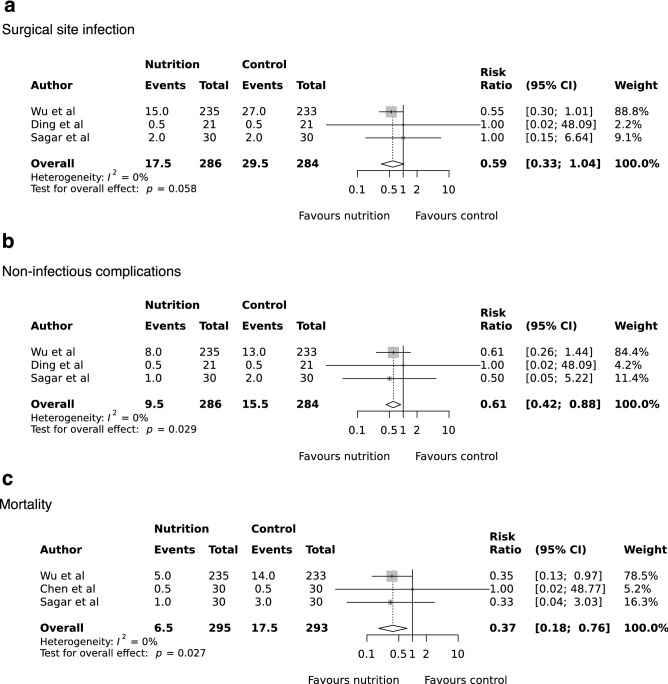
Random-effects meta-analysis of the effects of preoperative oral nutrition on surgical site infection (**a**), non-infectious complications (**b**), and mortality (**C**) in patients undergoing surgery for gastrointestinal cancer.

Three studies reported incidence of infectious complications (Fig. 3a)^21,22,27^. The pooled event rates for infectious complications were 14.3% (41/286) in the nutrition group and 27.8% (79/284) in the control arm. The pooled RR for infectious complications was 0.52 (95% CI 0.40 to 0.67, P = 0.008, *I*^2^ = 0%; Fig. 3a). The intervention had a consistently strong positive effect despite baseline rate heterogeneity across included studies (ARR range −0.20 to −0.07, pooled effect −0.13, 95% CI −0.22 to −0.06, P < 0.001; Fig. 3b), with a corresponding NNT of 8 (95% CI 5 to 14). Meanwhile the incidence of surgical site infection (SSI) was reported in three studies^21,22,27^, however none reported the criteria used to diagnose SSI. There were no SSI events up to 30 days postoperatively in one study^22^. SSI rates were 5.9% (17/286) in the nutrition group and 10.2% (29/284) in the control arm, with the pooled RR for SSI 0.59 (95% CI 0.33 to 1.04, P = 0.058, *I*^2^ = 0%; Fig. 4a).

Three studies reported the incidence of non-infectious complications^21,22,27^, with one study reporting no events at 30 days^22^. Pooled event rates were 3.1% (9/286) in the nutrition group and 5.3% (15/284) in the control arm. The pooled RR for non-infectious complications were 0.61 (95% CI 0.42 to 0.88, P = 0.029, *I*^2^ = 0%; Fig. 4b). Three studies reported on 30-day mortality^21,26,27^, with the pooled event rate 2.0% (6/295) in the nutrition group and 5.8% (17/293) in the control arm. The pooled RR for mortality was 0.37 (95% CI 0.18 to 0.76, P = 0.027, *I*^2^ = 0%; Fig. 4c).

### Assessment of bias

Publication bias was demonstrated to be low across all measured outcomes. The distribution of RR was evenly distributed across the funnel plot, with no significant outliers (Supplementary Fig. S4). The risk of bias for all included studies is summarised in Supplementary Fig. S5. Incomplete outcome data had a low risk of bias in all studies, with adequate sequence generation in five studies^21,22,24,26,27^. However the majority of other domains contained unclear or high risk, particularly for allocation concealment and outcome assessment.

## Discussion

This meta-analysis has demonstrated that the risks of infection, complications and all-cause mortality after surgery for gastrointestinal cancer in a LMIC setting were reduced in patients receiving preoperative nutrition. The intervention had a consistently strong effect on complication rates across each study population despite heterogeneity in baseline rates. However, the intervention did not impact upon SSI, with insufficient data available to assess length of stay, hospital costs, and return to work or household activity.

The analysis included patient populations from two countries, undergoing operations for gastrointestinal cancer. Statistical heterogeneity was found to be low for all outcomes measured and the risk of publication bias was also low. Overall treatment effects were robust during sensitivity analysis and due to methodology are likely to be conservative estimates. However, interventions were predominantly performed in China for patients undergoing surgery for gastric cancer and evidence of methodological bias was demonstrated.

Malnutrition is a major public health issue in LMICs and forms part of the United Nations 2030 Agenda for Sustainable Development^1^, reducing a patient’s ability to compensate for stressful events, such as major surgery^28^. While associated with poorer outcomes, malnutrition is potentially reversible^29^. The European Society for Parenteral and Enteral Nutrition (ESPEN) guidelines suggest nutritional support should be initiated without delay in patients undergoing surgery if oral intake reduction is expected 7 days perioperatively^30^, however the effectiveness of nutritional intervention in LMICs is uncertain.

Previously the ability of nutrition interventions to reduce infectious complications and length of hospital stay in a global population has been demonstrated^10^, however inclusion of high-income populations and parenteral routes limits generalizability to LMIC settings. A recent meta-analysis including only studies conducted in East Asia demonstrated no benefit of preoperative oral nutrition for postoperative complications^9^, however the sole inclusion of gastric cancer patients undergoing surgery, high weighting towards high-income country settings and significant study heterogeneity (58%) may explain differences with our findings.

Baseline rates for measured outcomes differed across studies, similar to variation demonstrated globally in large population cohorts across LMICs^31,32^. However, a consistently strong positive treatment effect was shown across measured outcomes, demonstrated by similar risk ratios and small confidence intervals. This suggests preoperative oral nutrition confers a positive effect independent of baseline complication rate and our findings are applicable across LMIC settings.

The method of administration was not always obvious and tube feeding may have been used in three studies^22–24^, yet sub-group analysis found treatment effects persisted following their exclusion. Some studies failed to report the formal criteria used to classify postoperative complications and SSI. Furthermore, disease stage, nutrition dose variation and potential unmeasured confounders will have introduced elements of clinical heterogeneity. However, the use of random-effects models, consistent treatment effects and low statistical heterogeneity overall supports our conclusions. Only four studies provided at least five days preoperative nutrition, in keeping with current guideline recommendations^33,34^. Therefore, our results may underestimate the overall effect of preoperative nutrition in LMICs.

Surgical site infection rates were low within included studies (overall rate 8.1%; range 0 – 12.6%), which suggests SSI may be under-reported^31^. The absence of definitive diagnostic criteria, such as those stated by the Centre for Disease Control and Prevention^35^, may explain this variation and the null effect of nutrition on SSI rates. In contrast, infectious complications reduced following preoperative nutrition, similar to another recent meta-analysis of immune modulating nutrition in high-income settings^8^.

Some limitations within our analysis exist. Cancer-focused studies commonly report longer-term survival, particularly at one and five years^36,37^, and the impact of preoperative oral nutrition on these outcomes remains unknown, with included studies only reporting data on short-term outcomes. However, the demonstrated absolute risk reduction in mortality may also influence longer-term survival in patients undergoing surgery for localised, potentially curative disease. Furthermore, the reduction in postoperative complications is likely to reduce delays to adjuvant treatment, which have been associated with worse survival^38–40^ and unfavourable oncological outcomes^41^ in a wide range of cancers.

Secondly, particular patient groups are under-represented within the meta-analysis. The effectiveness of interventions remains uncertain in some globally common malignancies, for example gynaecological and oral cancer^42^, and in a broader range of settings across Africa and the Asian subcontinent. More conclusive statements on the effectiveness of preoperative nutrition across LMICs is limited by the majority of studies conducted in China. Furthermore, despite being LMICs, advanced medical care is available in many parts of China and India. It is unclear if the circumstances of these included studies are truly representative of the challenges other countries might face with implementation, and the populations they will be predominantly treating. Lastly, the majority of interventions were commercially sourced, with no cost-effectiveness data to support this strategy within LMIC settings. It remains to be demonstrated whether nutritionally balanced, locally sourced low-cost supplements would be as effective.

If these research gaps are addressed, preoperative oral nutrition is likely to form part of future global surgical guidelines as a simple measure that can improve outcomes after surgery for cancer. Planned trials should particularly focus on determining the impact of oral nutrition in Africa and the Asian subcontinent, with at least one expected in the near future^43^.

## Conclusion

This meta-analysis provides substantial evidence that preoperative oral nutrition in patients undergoing surgery for gastrointestinal cancer has a significant impact on postoperative complications and all-cause mortality. Treatment effects remained consistent despite variation in baseline complication rates and suggest generalisability across income strata. However, high quality randomised control trials across a wider LMIC surgical population are required to validate our findings based on current low to moderate quality of evidence.

## Supplementary Information

Supplementary Information.

## Data Availability

All data included within the meta-analysis is freely available within the public domain as all studies are published. The search strategy is available in the Supplementary material, and any additional data are available on reasonable request to the corresponding author.

## References

[1] Sustainable Development Goals. Sustainable Development Knowledge Platform. https://sustainabledevelopment.un.org/?menu=1300.

[2] Nakahara S (2017). Perioperative nutrition management as an important component of surgical capacity in low- and middle-income countries. Trop. Med. Int. Health.

[3] Shpata V (2014). Malnutrition at the time of surgery affects negatively the clinical outcome of critically ill patients with gastrointestinal cancer. Med. Arch..

[4] Waitzberg DL, Caiaffa WT, Correia MI (2001). Hospital malnutrition: The Brazilian national survey (IBRANUTRI): a study of 4000 patients. Nutrition.

[5] Nepogodiev D (2019). Prioritizing research for patients requiring surgery in low- and middle-income countries. BJS (British Journal of Surgery).

[6] Meara JG, Hagander L, Leather AJM (2014). Surgery and global health: A Lancet Commission. Lancet.

[7] Sullivan R (2015). Global cancer surgery: Delivering safe, affordable, and timely cancer surgery. Lancet Oncol..

[8] Adiamah A, Skořepa P, Weimann A, Lobo DN (2019). The impact of preoperative immune modulating nutrition on outcomes in patients undergoing surgery for gastrointestinal cancer: A systematic review and meta-analysis. Ann. Surg..

[9] Chen X, Yang K, Zhang X, Li K (2019). Meta-analysis of preoperative oral nutritional supplements for patients with gastric cancer: East Asian experience. Eur. J. Clin. Nutr..

[10] Zhong J, Kang K, Shu X (2015). Effect of nutritional support on clinical outcomes in perioperative malnourished patients: A meta-analysis. Asia Pac. J. Clin. Nutr..

[11] PROSPERO: International prospective register of systematic reviews. [Accessed Jun, 3 2022] 10.1186/2046-4053-1-2 PMC3348673 22587842

[12] Moher D, Liberati A, Tetzlaff J, Altman DG, Group TP (2009). Preferred reporting items for systematic reviews and meta-analyses: The PRISMA Statement. PLOS Med..

[13] Cochrane LMIC Filters for PubMed (NLM), MEDLINE (Ovid), Embase (Ovid), and CENTRAL (Cochrane Library) to help identify studies relevant to LMIC. https://epoc.cochrane.org/lmic-filters. [Accessed Jun, 3 2022]

[14] Covidence - Better systematic review management. https://www.covidence.org/home. [Accessed Jun, 3 2022]

[15] World Bank Country and Lending Groups – World Bank Data Help Desk. https://datahelpdesk.worldbank.org/knowledgebase/articles/906519. [Accessed Jun, 3 2022]

[16] Knight SR (2019). Systematic review of the use of big data to improve surgery in low- and middle-income countries. Br J Surg.

[17] Higgins JPT (2011). The Cochrane Collaboration’s tool for assessing risk of bias in randomised trials. BMJ.

[18] Cochrane Handbook for Systematic Reviews of Interventions. https://handbook-5-1.cochrane.org/. [Accessed Jun, 3 2022]

[19] Friedrich JO, Adhikari NK, Beyene J (2007). Inclusion of zero total event trials in meta-analyses maintains analytic consistency and incorporates all available data. BMC Med. Res. Methodol..

[20] Viechtbauer W, Cheung MW-L (2010). Outlier and influence diagnostics for meta-analysis. Res. Synth. Methods.

[21] Wu G-H, Liu Z-H, Wu Z-H, Wu Z-G (2006). Perioperative artificial nutrition in malnourished gastrointestinal cancer patients. World J. Gastroenterol..

[22] Ding G, Chen P, Yi Z, Zheng Q (2009). Roles of nutrition risk screening and preventive enteral nutritional support before radical resection of gastric cancer. Chin. J. Gastrointest. Surg..

[23] Zheng Q, Chen P, Ding G (2010). Significance of preoperative shortterm preventive enteral nutrition support in patients with gastric cancer who are at risk of malnutrition. Mod. Pract. Med..

[24] Kharbuja, P. Efficacy of preoperative nutritional supports on postoperative outcome in gastric cancer patients at nutritional risk by NRS-2002: A prospective, randomized clinical trial. Jilin University (2013).

[25] Zhou, L. Influences of preoperative enteral nutrition combined with probiotics on the clinical outcomes in postoperative gastric cancer patients. Nanchang University (2016).

[26] Chen J, Ye J, Song W, He Y (2013). Application of enteral nutrition in preoperative bowel preparation for rectal cancer patients undergoing radical operation. Zhonghua Wei Chang Wai Ke Za Zhi.

[27] Sagar RC (2019). Perioperative artificial enteral nutrition in malnourished esophageal and stomach cancer patients and its impact on postoperative complications. Indian J. Surg. Oncol..

[28] Sungurtekin H, Sungurtekin U, Balci C, Zencir M, Erdem E (2004). The influence of nutritional status on complications after major intraabdominal surgery. J. Am. Coll Nutr.

[29] Allison SP (2000). Malnutrition, disease, and outcome. Nutrition.

[30] Weimann A (2017). ESPEN guideline: Clinical nutrition in surgery. Clin. Nutr..

[31] Collaborative GlobalSurg (2018). Surgical site infection after gastrointestinal surgery in high-income, middle-income, and low-income countries: A prospective, international, multicentre cohort study. Lancet Infect. Dis..

[32] Collaborative GlobalSurg (2016). Mortality of emergency abdominal surgery in high-, middle- and low-income countries. Br. J. Surg..

[33] Williams J, Wischmeyer P (2017). Assessment of perioperative nutrition practices and attitudes—A national survey of colorectal and GI surgical oncology programs. Am. J. Surg..

[34] McClave SA (2013). Summary points and consensus recommendations from the North American Surgical Nutrition Summit. J. Parenter. Enteral Nutr..

[35] Surgical Site Infection | Guidelines | Infection Control | CDC. https://www.cdc.gov/infectioncontrol/guidelines/ssi/index.html. [Accessed Jun, 3 2022]

[36] Arnold M (2019). Progress in cancer survival, mortality, and incidence in seven high-income countries 1995–2014 (ICBP SURVMARK-2): A population-based study. Lancet Oncol..

[37] Allemani C (2018). Global surveillance of trends in cancer survival 2000–14 (CONCORD-3): Analysis of individual records for 37 513 025 patients diagnosed with one of 18 cancers from 322 population-based registries in 71 countries. Lancet.

[38] Gao P (2018). Impact of timing of adjuvant chemotherapy on survival in stage III colon cancer: A population-based study. BMC Cancer.

[39] Perez CA, Grigsby PW, Castro-Vita H, Lockett MA (1995). Carcinoma of the uterine cervix. I. Impact of prolongation of overall treatment time and timing of brachytherapy on outcome of radiation therapy. Int. J. Radiat. Oncol. Biol. Phys..

[40] Ma SJ (2019). Association of timing of adjuvant therapy with survival in patients with resected stage I to II pancreatic cancer. JAMA Netw Open.

[41] Noh GT (2020). The impact of early adjuvant chemotherapy in rectal cancer. PLoS ONE.

[42] Bray F (2018). Global cancer statistics 2018: GLOBOCAN estimates of incidence and mortality worldwide for 36 cancers in 185 countries. CA Cancer J. Clin..

[43] CRANE Feasibility Study: Nutritional Intervention for Patients Undergoing Cancer Surgery in Low- and Middle-Income Countries - Full Text View - ClinicalTrials.gov. https://clinicaltrials.gov/ct2/show/NCT04448041. [Accessed Jun, 3 2022]

