# Supplementary material for: The impact of preoperative oral nutrition supplementation on outcomes in patients undergoing gastrointestinal surgery for cancer in low- and middle-income countries: a systematic review and meta-analysis
Source: Sci Rep. 2022 Jul 21;12:12456. doi: 10.1038/s41598-022-16460-4 (PMC9304351; doi:10.1038/s41598-022-16460-4)
Supplement: Supplementary file 1 — Supplementary Information. [file 41598_2022_16460_MOESM1_ESM.pdf]

Supplementary material. Systematic review literature search terms

EMBASE/Medline

1. Developing Countries.sh,kf.
2. (Africa or Asia or Caribbean or West Indies or South America or Latin America or Central America).hw,kf,ti,ab,cp.
3. (Afghanistan or Albania or Algeria or Angola or Armenia or Armenian or Azerbaijan or Bangladesh or Benin or Byelarus or Byelorussian or Belarus or Belorussian or Belorussia or Belize or Bhutan or Bolivia or Bosnia or Herzegovina or Hercegovina or Botswana or Brasil or Brazil or Bulgaria or Burkina Faso or Burkina Fasso or Upper Volta or Burundi or Urundi or Cambodia or Khmer Republic or Kampuchea or Cameroon or Cameroons or Cameron or Camerons or Cape Verde or Central African Republic or Chad or China or Colombia or Comoros or Comoro Islands or Comores or Mayotte or Congo or Zaire or Costa Rica or Cote d'Ivoire or Ivory Coast or Croatia or Cuba or Cyprus or Djibouti or French Somaliland or Dominica or Dominican Republic or East Timor or East Timur or Timor Leste or Ecuador or Egypt or United Arab Republic or El Salvador or Eritrea or Ethiopia or Fiji or Gabon or Gabonese Republic or Gambia or Gaza or Georgia Republic or Georgian Republic or Ghana or Gold Coast or Greece or Grenada or Guatemala or Guinea or Guam or Guiana or Guyana or Haiti or Honduras or India or Maldives or Indonesia or Iran or Iraq or Jamaica or Jordan or Kazakhstan or Kazakh or Kenya or Kiribati or Kosovo or Kyrgyzstan or Kirghizia or Kyrgyz Republic or Kirghiz or Kirgizstan or Lao PDR or Laos or Lebanon or Lesotho or Basutoland or Liberia or Libya or Lithuania or Macedonia or Madagascar or Malagasy Republic or Sabah or Sarawak or Malawi or Nyasaland or Mali or Marshall Islands or Mauritania or Mauritius or Agalega Islands or Mexico or Micronesia or Middle East or Moldova or Moldovia or Moldovian or Mongolia or Montenegro or Morocco or Ifni or Mozambique or Myanmar or Myanma or Burma or Namibia or Nepal or Netherlands Antilles or New Caledonia or Nicaragua or Niger or Nigeria or Northern Mariana Islands or Oman or Muscat or Pakistan or Palau or Palestine or Paraguay or Peru or Philippines or Philipines or Phillipines or Phillippines or Romania or Rumania or Roumania or Russia or Russian or Rwanda or Ruanda or Saint Kitts or St Kitts or Nevis or Saint Lucia or St Lucia or Saint Vincent or St Vincent or Grenadines or Samoa or Samoan Islands or Navigator Island or Navigator Islands or Sao Tome or Senegal or Serbia or Montenegro or Seychelles or Sierra Leone or Slovenia or Sri Lanka or Ceylon or Solomon Islands or Somalia or South Africa or Sudan or Suriname or Surinam or Swaziland or Syria or Tajikistan or Tadzhikistan or Tadjikistan or Tadzhik or Tanzania or Thailand or Togo or Togolese Republic or Tonga or Trinidad or Tobago or Tunisia or Turkey or Turkmenistan or Turkmen or Uganda or Ukraine or USSR or Soviet Union or Union of Soviet Socialist Republics or Uzbekistan or Uzbek or Vanuatu or New Hebrides or Venezuela or Vietnam or Viet Nam or West Bank or Yemen or Yugoslavia or Zambia or Zimbabwe or Rhodesia).hw,ti,ab,cp.
4. ((developing or less* developed or under developed or underdeveloped or middle income or low* income or underserved or under served or deprived or poor*) adj (countr* or nation? or population? or world)).ti,ab.
5. ((developing or less* developed or under developed or underdeveloped or middle income or low* income) adj (economy or economies)).ti,ab.
6. (low* adj (gdp or gnp or gross domestic or gross national)).ti,ab.
7. (low adj3 middle adj3 countr*).ti,ab.
8. (lmic or lmics or third world or lami countr*).ti,ab.
9. transitional countr*.ti,ab.
10. or/1-9
11. exp surgery/ or surg* or cancer/ or canc* or malignancy or malignan* or oncolog*
12. exp nutrition/ or nutri* or diet/ or food
13. outcome or intervention or interve* or trial
14. and/10-13
15. Limits: Humans, all adult (19 plus years)

Supplementary Table S1. Summary of inclusion and exclusion criteria

**Parameter**	**Inclusion criteria**	**Exclusion criteria**
Patients	Adult patients (≥18 years old) undergoing surgery for gastrointestinal cancer in LMICs	Surgery for benign disease or non-gastrointestinal cancer or patients treated in high-income countries
Intervention	Preoperative oral nutritional supplement (ONS) containing macronutrients (fat, carbohydrate and protein) with or without micronutrients (vitamins and minerals)	Nutritional intervention using single nutrient substrates, complementary food substances, probiotic formulas or as part of a multimodal preoperative intervention (such as an enhanced recovery programme) and those delivered by enteral tubes or parenteral routes. Postoperative nutritional intervention.
Comparator	Patients receiving routine care with no additional dietary supplementation	
Outcomes	At least one clinical outcome (complication rate, mortality, surgical site infection, anastomotic leak)	
Study design	Randomised controlled trials	Non-randomised controlled trials

LMIC: low- and middle-income countries

Supplementary Table S2. Summary of nutritional interventions and outcomes measured

	Year	Control	Oral nutritional intervention used	Intervention commenced preoperatively (days)	Length of intervention (days)	Outcomes measured
Wu et al	2006	Standard diet	25 kcal/kg/day non-protein and 0·25g nitrogen/kg/day	7	14	Post-operative complications, SSI, mortality, length of stay
Ding et al	2009	Standard diet	Nutrison® Liquid 1000ml (1 kcal/ml)	3	3	Post-operative complications, SSI, weight, bloods, Immunoglobulin
Zheng et al	2010	Standard diet	Nutrison® Liquid 500ml (1 kcal/ml)	3	3	Post-operative complications, bloods, Immunoglobulin
Kharbuja et al	2013	Standard diet	Nutrison® Liquid 500ml (1 kcal/ml)	5-7	Up to 7	Post-operative complications
Chen et al	2013	Standard diet	Whole protein enteral nutrition	3	3	Post-operative complications, mortality, bloods
Zhou et al	2016	Standard diet	Nutrison® Powder 320g (1 kcal/ml)	7	7	Post-operative complications, bloods, Immunoglobulin
Sagar et al	2019	Routine diet	25 kcal/kg/day non-protein and 0·25 g/kg/day nitrogen	15-20	15	Post-operative complications, SSI, mortality, length of stay

SSI – Surgical site infection

Supplementary Table S3. Summary of pooled results

	Study number	Patients	Pooled treatment effect RR (95% CI)
All complications	7	891	0·53 (0·46 to 0·60)
Infectious complications	3	570	0·52 (0·40 to 0·67)
Surgical site infection	3	570	0·59 (0·33 to 1.04)
Non-infectious complications	3	570	0·61 (0·42 to 0·88)
Mortality	3	588	0·37 (0·18 to 0·76)

All pooled treatment effects calculated using Mantel-Haenszel random-effects models. RR – Risk ratio

Supplementary Figure S1. Random-effects meta-analysis of the effects of preoperative oral nutrition on all postoperative complications, excluding studies using Nutrison® liquid

	Nutrition		Control				
**Author**	**Events**	**Total**	**Events**	**Total**	**Risk Ratio**	**(95% CI)**	**Weight**
Wu et al	43	235	78	233	0.55	[0.39; 0.76]	68.0%
Chen et al	5	30	6	30	0.83	[0.28; 2.44]	9.3%
Zhou et al	3	20	6	20	0.50	[0.14; 1.73]	7.0%
Sagar et al	6	30	14	30	0.43	[0.19; 0.96]	15.7%
**Overall**	**57**	**315**	**104**	**313**	**0.54**	**[0.40; 0.73]**	**100.0%**

Heterogeneity: $I^2 = 0\%$
Test for overall effect: $p = 0.007$

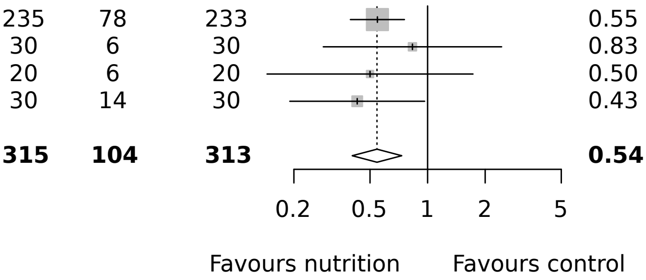

Supplementary Figure S2. Influence analysis (A) and graphic display of heterogeneity (GOSH) plot (B) of included studies exploring potential presence of heterogeneity and sub-clusters with different effect sizes for all complications

a

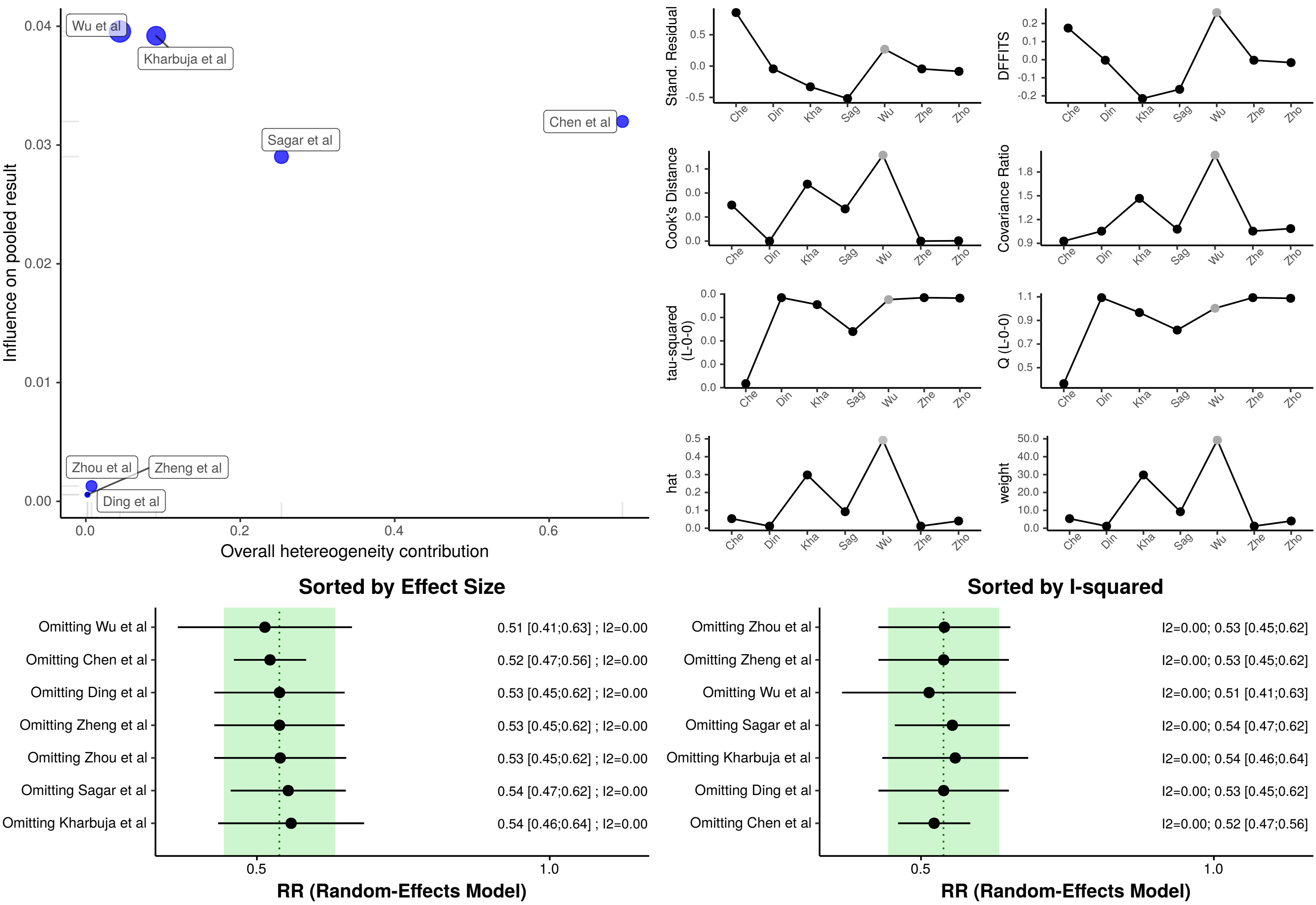

b

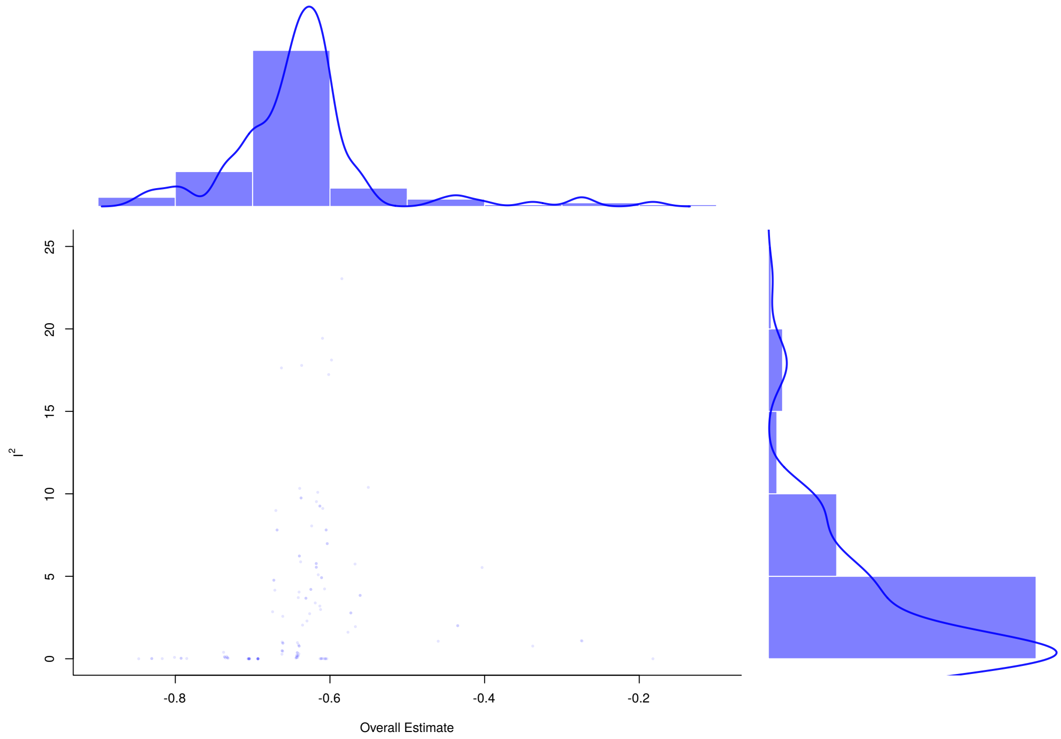

Supplementary Figure S3. Forest plot demonstrating sensitivity analysis for all complications between preoperative oral nutritional intervention and control groups excluding Wu et al. (2006).

Author	Nutrition Events	Nutrition Total	Control Events	Control Total	Risk Ratio	(95% CI)	Weight
Ding et al	1	21	2	21	0.50	[0.05; 5.10]	2.3%
Zheng et al	1	18	2	18	0.50	[0.05; 5.04]	2.4%
Kharbuja et al	21	92	43	93	0.49	[0.32; 0.76]	58.1%
Chen et al	5	30	6	30	0.83	[0.28; 2.44]	10.7%
Zhou et al	3	20	6	20	0.50	[0.14; 1.73]	8.1%
Sagar et al	6	30	14	30	0.43	[0.19; 0.96]	18.4%
**Overall**	**37**	**211**	**73**	**212**	**0.51**	**[0.41; 0.63]**	**100.0%**

Heterogeneity: $I^2 = 0\%$
Test for overall effect: $p < 0.001$

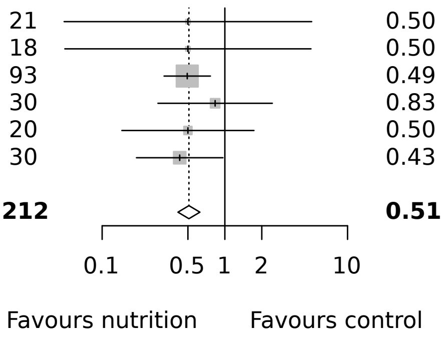

Supplementary Figure S4. Publication bias across all measured outcomes; all complications (A), infectious complications (B), surgical site infection (C), non-infectious complications (D), and mortality (E)

a

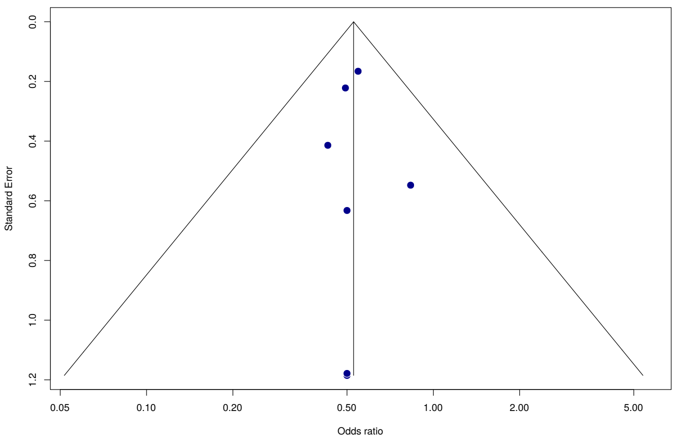

Egger’s test: 0.026 (-0.562 to 0.614); p = 0.934

b

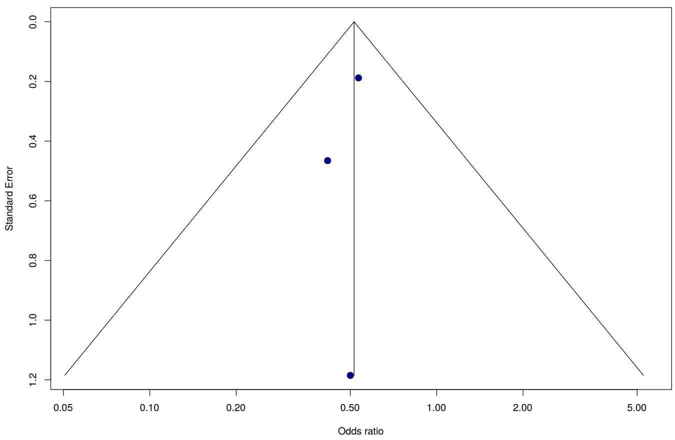

Egger’s test: -0.310 (-1.09 to 0.474); p = 0.582

c

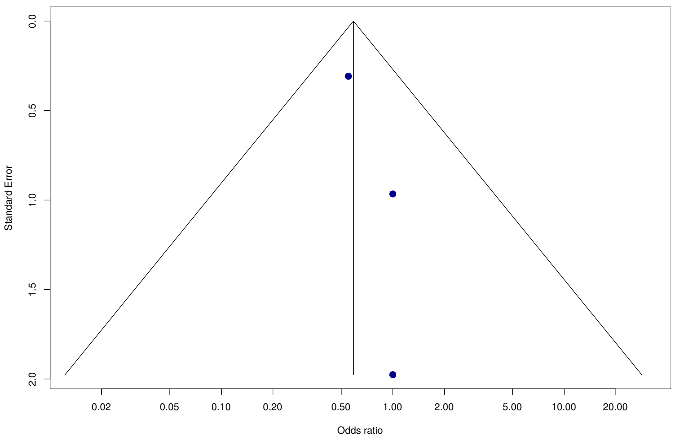

Egger’s test: 0.561 (-0.027 to 1.149); p = 0.293

d

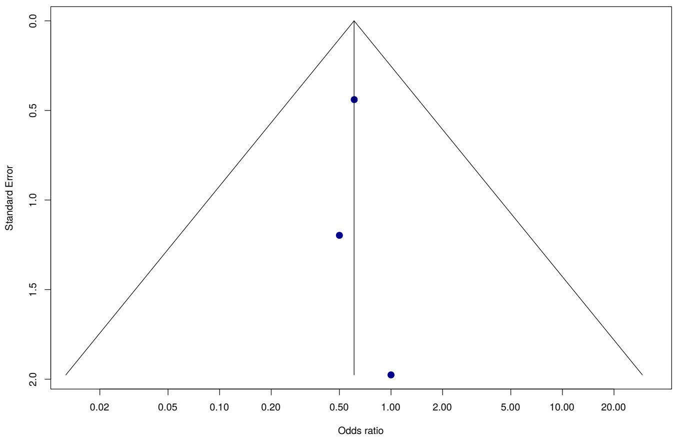

Egger’s test: 0.105 (-0.483 to 0.693); p = 0.789

e

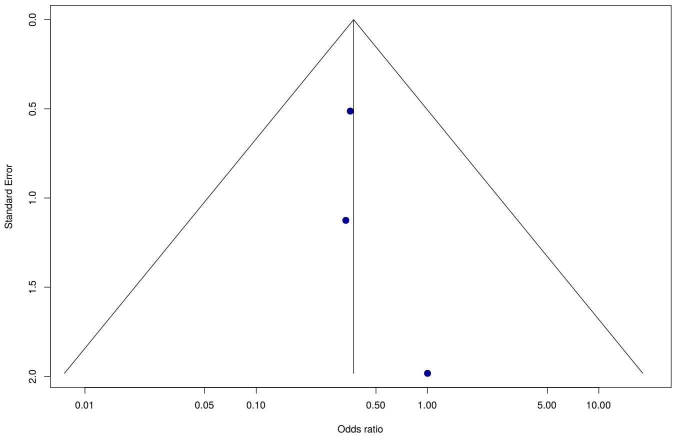

Egger’s test: 0.462 (-0.322 to 1.246); p = 0.466

Supplementary Figure S5. Risk of bias summary (A) and individual assessment (B) for included randomised controlled trials

a

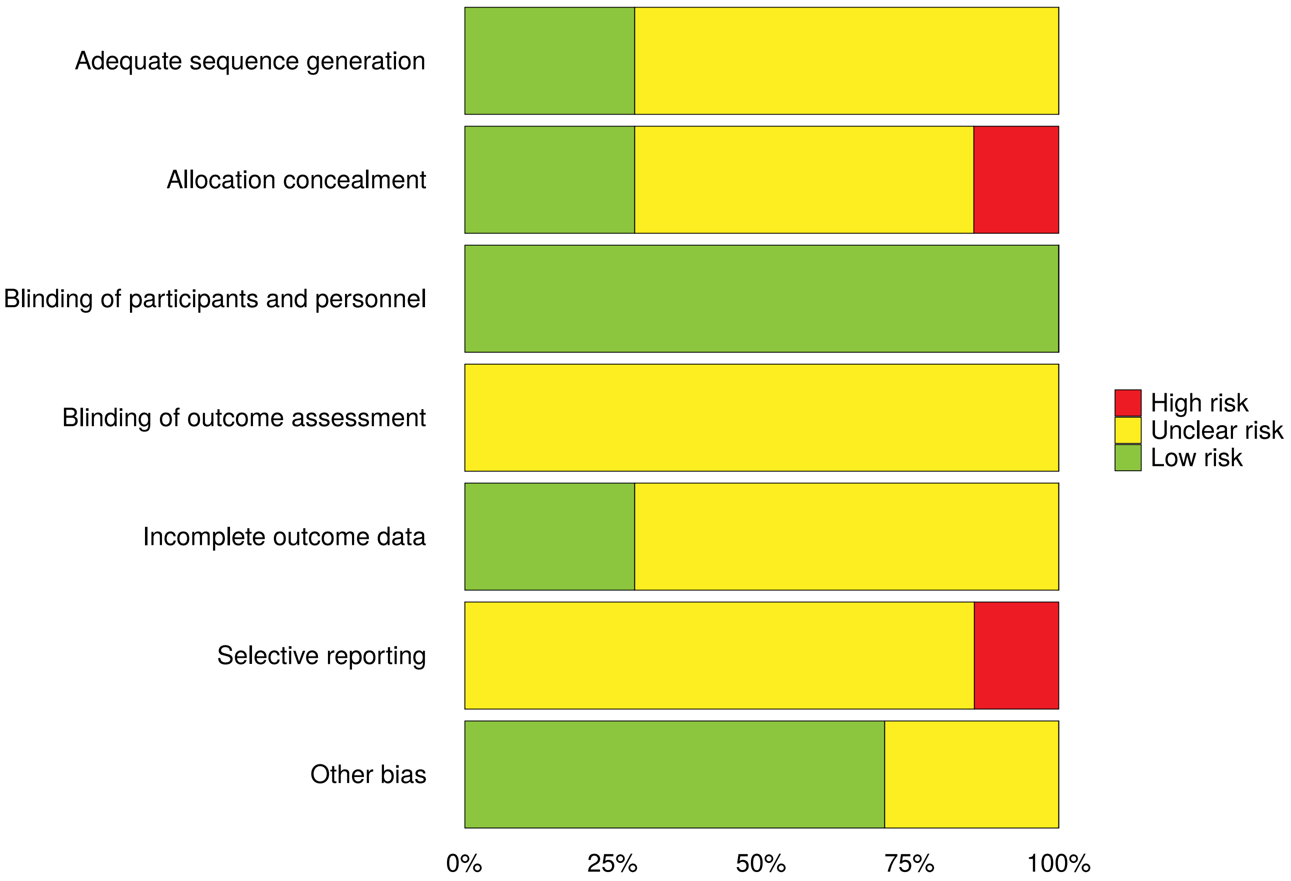

b

	Adequate sequence generation	Allocation concealment	Blinding of participants and personnel	Blinding of outcome assessment	Incomplete outcome data	Selective reporting	Other bias
Wu et al 2006	+	?	+	?	+	+	+
Ding et al 2009	+	?	?	?	+	?	?
Zheng et al 2010	?	?	?	?	+	?	?
Chen et al 2013	+	−	?	?	+	−	?
Kharbuja et al 2015	+	?	?	?	+	?	?
Zhou et al 2016	?	?	?	?	+	?	?
Sagar et al 2019	+	?	+	?	+	+	+

Key

+ Low risk of bias

? Unclear risk of bias

− High risk of bias
